# Evaluation of Antioxidant and Antibacterial Activity of Gelatin Nanoparticles with Bitter Orange Peel Extract for Food Applications

**DOI:** 10.3390/foods13233838

**Published:** 2024-11-28

**Authors:** Adamaris García-Juárez, Alba Mery Garzón-García, José Rogelio Ramos-Enríquez, José Agustín Tapia-Hernández, Saúl Ruiz-Cruz, Dalila Fernanda Canizales-Rodríguez, Carmen Lizette Del-Toro-Sánchez, Francisco Rodríguez-Félix, Víctor Manuel Ocaño-Higuera, José de Jesús Ornelas-Paz

**Affiliations:** 1 Departamento de Investigación y Posgrado en Alimentos, Universidad de Sonora, Encinas y Rosales s/n, Hermosillo 83000, Sonora, Mexico; a218203582@unison.mx (A.G.-J.); joseagustin.tapia@unison.mx (J.A.T.-H.); carmen.deltoro@unison.mx (C.L.D.-T.-S.); francisco.rodriguezfelix@unison.mx (F.R.-F.); 2 Departamento de Ciencias Químico Biológicas, Universidad de Sonora, Encinas y Rosales s/n, Hermosillo 83000, Sonora, Mexico; rogelio.ramos@unison.mx (J.R.R.-E.); dalila.canizales@unison.mx (D.F.C.-R.); victor.ocano@unison.mx (V.M.O.-H.); 3 Tecnología Agroambiental, Universidad del Valle Sede Regional Caicedonia, Carrera 14 No 4-48, Caicedonia 76250, Valle del Cauca, Colombia; garzon.alba@correounivalle.edu.co; 4 Facultad de Ingeniería y Administración, Universidad Nacional de Colombia—Sede Palmira, Carrera 32 # 12-00, Palmira 763533, Valle del Cauca, Colombia; 5 Centro de Investigación en Alimentación y Desarrollo, Av. Río Conchos S/N Parque Industrial, Cuauhtémoc 31570, Chihuahua, Mexico; jornelas@ciad.mx

**Keywords:** *Citrus aurantium*, nanotechnology, coaxial electrospray, bioactive compounds, foodborne pathogens

## Abstract

Bitter orange is a citrus fruit rich in bioactive compounds, but its waste is currently underutilized. One potential solution is to encapsulate these bioactive compounds. This research aims to synthesize gelatin nanoparticles loaded with an ethanolic extract of bitter orange peel and to evaluate their in vitro antioxidant and antibacterial activities. Coaxial electrospray was used to encapsulate the ethanolic extract of bitter orange with bovine gelatin as wall material, considering a voltage of 15 kV, a wall solution flow rate of 0.1 mL/h, and a core solution flow rate of 0.08 mL/h. Characterization of the nanoparticles was performed using scanning electron microscopy (SEM) and Fourier transform infrared spectroscopy (FT-IR). Antioxidant activity was evaluated by the total phenolic content, flavonoids, and antioxidant capacity by the DPPH^•^, ABTS^•+^, and FRAP assays. Antibacterial activity was assessed by the well diffusion technique on Mueller–Hinton agar against *Listeria monocytogenes* and *Escherichia coli* O157:H7 bacteria. SEM images confirmed that the nanoparticles were spherical in shape, while FT-IR analysis indicated that the incorporation of the extract did not alter the amide bonds of the gelatin protein. The nanoparticles containing the extract exhibited higher antioxidant activity and heightened inhibition against *E. coli* O157:H7, indicating their potential food applications.

## 1. Introduction

**Figure 1:**
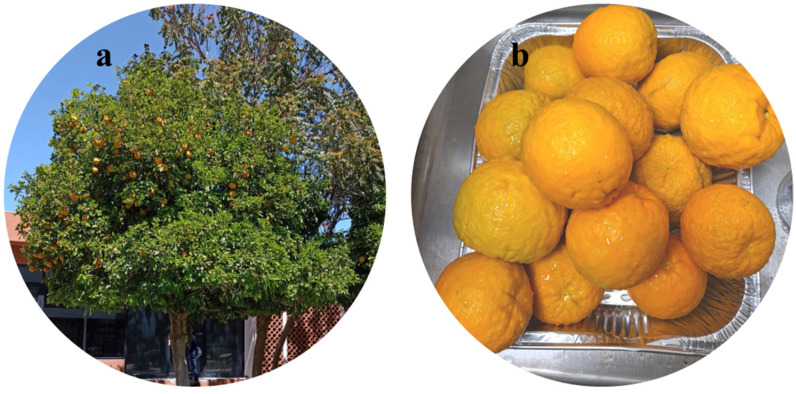
Tree (**a**) and fruit (bitter orange) (**b**) of the *Citrus aurantium* species.

The bitter orange (*Citrus aurantium*) is a fruit that grows on a tree belonging to the *Rutaceae* family. Native to East Africa, Arabia, and Syria, it is actively cultivated in regions such as Spain, Italy, and North America [1]. In Sonora, Mexico, *Citrus aurantium* is primarily used as an ornamental tree, and its fruits are typically discarded [2]. Bitter orange is a globose fruit with a slightly textured surface and an intense orange color when ripe (Figure 1). Due to its rich content of diverse bioactive compounds such as phenolics, flavonoids, essential oils, and vitamins, bitter orange is commonly employed for medicinal purposes (antibacterial, anti-inflammatory, anticancer, antioxidant, and cardiovascular properties) and in the food industry (juices and essential oils) [3]. Bitter orange biowaste currently produces atmospheric and aquatic pollution contamination since it accumulates in the soil when it is not consumed or it is not fully used [4]. The predominant residue of the bitter orange is its peel, comprising approximately 40% of its fresh weight. In the peel, the documented phenolic compounds primarily consist of phenolic acids (74%) and flavonoids (23%). Among these, the most prevalent phenolic compounds identified are p-coumaric acid and ferulic acid [5].

The previously mentioned compounds can help prevent food spoilage and cross-contamination, which pose significant health risks to consumers and can lead to temporary digestive issues such as gastroenteritis, diarrhea, fever, or even infections in other systems. Bacteria such as *Escherichia coli* and *Listeria monocytogenes* are often found in raw or poorly processed foods, including meat, dairy products, seafood, and fruits, making them two of the primary etiological agents of foodborne diseases [6]. There is currently a growing interest in obtaining bioactive compounds from biowaste due to their antioxidant and antimicrobial properties. These compounds also modulate the intestinal microbiota and promote the immune system [2]. Biowaste is rich in phenolic molecules, which contain aromatic rings with hydroxyl groups. These molecules are known to inhibit microorganisms by interacting with their cytoplasmic membranes, cell walls, and nucleic acids, thereby disrupting cellular functions, slowing growth, and causing cell death [7,8].

Nanoencapsulation is a process in which an active ingredient, an active molecule of a food (antioxidants, essential fatty acids, vitamins), or living cells (probiotics) are trapped within a wall material (polymers of carbohydrates, proteins, and lipids). Encapsulation serves not only to enhance the stability, bioavailability, and controlled release characteristics of biomolecules but also aids in concealing undesirable odor and taste [9]. Different nanoencapsulation techniques exist, notable among them are nanoprecipitation, emulsion–diffusion, double-emulsification, emulsion–coacervation, polymer coating, and electrospray techniques. However, electrospray is relatively simple, low cost, requires a low amount of solvents and the particles are obtained in a single step [10]. The principle of electrospray lies in the ability of an electric field to deform the droplet interface to produce nanometer-range droplets. The Rayleigh method serves to determine the rupture of the drop. This limit occurs when the surface tension of the drop is elevated by the electrostatic force [11]. The electrospray control variables, such as solution flow rate, electric potential, collector distance, environmental humidity, and polymer solution viscosity, conductivity, surface tension, and concentration, must be considered [12]. On the other hand, coaxial electrospray modifies the uniaxial electrospray process by using a coaxial capillary needle and syringes for two solutions [13]. It is an electrohydrodynamic process in which multilayer nanoparticles are formed by introducing coaxial electrified jets. Coaxial electrospray offers several potential benefits, including high efficiency, a simple and cost-effective process, the ability to preserve biological activity, and the production of particles with a uniform size distribution [12]. Therefore, the objective of this research is to produce by coaxial electrospray gelatin nanoparticles containing an ethanolic extract of bitter orange peel (*Citrus aurantium*) and to analyze their morphological, structural, antioxidant, and antibacterial properties as potential food applications.

## 2. Materials and Methods

### 2.1. Plant Material

Bitter orange fruits were harvested in the first quarter of 2022 at the University of Sonora, Hermosillo, Sonora, Mexico. A total of 900 g of fruit was washed with a neutral detergent, rinsed with potable water, and the peel was removed. The orange peel was cut into 3 cm by 3 cm squares and dried for 24 h at 60 °C in a convection oven (Blue M, New Columbia, PA, USA) until it reached a moisture content of 17%. Following drying, the material underwent grinding and sieving (size 60 sieve).

### 2.2. Chemical and Reagents

Absolute ethanol (≥99.5%) and hydrochloric acid (HCl, 37.5%) were from Meyer Reagents (Ciudad de México, MEX); gallic acid, 2 N Folin solution, quercetin, 2,2-diphenyl-1-picrylhydrazyl (DPPH^•^) radical, 6-hydroxy-2,5,7,8-tetramethylchroman-2-carboxylic acid (Trolox), tripyridyl-s-triazin (TPTZ), 2,2′-azino-bis(3-ethylbenzothiazoline-6-sulfonic acid) (ABTS^•+^) radical, glacial acetic acid, and bovine gelatin type B were obtained from Sigma-Aldrich (St. Louis, MO, USA); potassium persulfate (K_2_S_2_O_8_, ≥99.0%) was from Jalmek (San Nicolás de los Garza, NL, MEX); anhydrous sodium carbonate (Na_2_CO_3_, ≥99.5%), anhydrous sodium acetate (C_2_H_3_NaO_2_, ≥99.0%), and iron trichloride (FeCl_3_) were procured from CTR SCIENTIFIC (Monterrey, NL, MEX); Tryptic Soy Broth and Oxford Agar were purchased from MCD LAB (Tlaneplanta de Baz, EM, MEX); MacConkey Sorbitol Agar and Mueller–Hinton Agar were from Difco (Sparks, MD, USA); Ultrapure water was obtained using a purification system (Merck, Darmstadt, HE, GER).

### 2.3. Preparation of the Extract of Bitter Orange Peel

The initial solutions were prepared with 1.5 g of orange peel and 10 mL of absolute ethanol. These solutions were then homogenized, subjected to sonication for 20 min, and subsequently centrifuged at 1500 rpm for 15 min (Compact II Centrifuge, BD, East Rutherford, NJ, USA). The supernatants were collected, and the residues underwent a second extraction [14]. Thus, the supernatants were combined and concentrated with the rotary evaporator R-100 V (Flawil, SG, CH) at 200 rpm, 40 °C, and 90 kP. The concentrated samples were suspended in ethanol until a known concentration was obtained. This process was conducted in two batches.

### 2.4. Preliminary Test for Nanoencapsulation

Uniaxial electrospray was used to test the production of gelatin nanoparticles [15,16]. For the feed solution, a concentration of 10% *w*/*v* gelatin was considered. This solution was prepared with 20% aqueous acetic acid, then sealed with parafilm, and stirred under ambient conditions until complete dissolution was achieved, approximately 1 h. In the electrospray process, a flow rate in the pump (KD Scientific, Holliston, MA, USA) of 0.1 mL/h was maintained for the polymer solution. The voltage applied using a CZE 1000R high-voltage power supply (Spellman, Hauppauge, NY, USA) was 15 kV, and the distance to the collector varied from 5, 10, and 15 cm. The distance to produce the gelatin nanoparticles was selected based on the lowest mean diameter (MD) and polydispersity index (PI). MD was measured using ImageJ 1.53 k (Wayne Rasband, Bethesda, MD, USA), while PI was calculated according to Estrella-Osuna et al. [17] and a frequency analysis.

(1) $$PI=\sigma /\bar{X},$$

where σ corresponds to standard deviation $\bar{X}$ refers to the mean diameter of nanoparticles.

### 2.5. Preparation of Gelatin Nanoparticles Containing an Extract of Bitter Orange Peel

The coaxial electrospray process was conducted considering the optimal distance for gelatin nanoparticle production, a voltage of 15 kV, a flow rate of 0.1 mL/h for the polymer solution (outer layer material), and a flow rate of 0.08 mL/h for the extract of bitter orange peel, which was the inner material.

### 2.6. Morphological and Structural Characterization of Nanoparticles

The morphological characterization of the nanoparticles was conducted by scanning electron microscopy (SEM) using a JEOL 5410 LV equipment (Arkishima, Tokyo, Japan). An acceleration voltage of up to 15 kV and samples were positioned onto double-sided conductive carbon tape affixed to the aluminum pin stub of the SEM [18].

The infrared spectrum was obtained using an FT-IR spectrophotometer (Frontier, Perkin Elmer, Waltham, MA, USA). The spectra were recorded using the attenuated total reflectance (ATR) technique in transmittance mode. A spectrum scan from 4000 to 400 cm^−1^ was considered [19]. The determinations were performed three times.

### 2.7. Preparation of Nanoparticle Suspension

The empty nanoparticles and those that included the ethanolic extract of bitter orange peel were suspended in absolute ethanol for approximately 12 h. Subsequently, these solutions were centrifuged at 1500 rpm for 15 min (COMPACT II CENTRIFUGE, BD, NJ, USA). The supernatants at a concentration of 2000 μg/mL were used to determine the antioxidant and antibacterial activity.

### 2.8. Determination of Total Phenolic and Flavonoids Contents

The total phenolic content was determined in accordance with Garzón-García et al. [20]. A total of 10 μL of samples (extract of bitter orange peel, solutions of empty gelatin nanoparticles, and nanoparticles containing the extract) and 25 μL of 1 N Folin solution were added to the microplate and refrigerated for 5 min. After refrigeration, 25 μL of 20% Na_2_CO_3_ and 140 μL of distilled water were added to the wells. The microplate was put to rest for 30 min, and the absorbance was measured at 760 nm (Multiskan GO, Thermo Fisher Scientific, Waltham, MA, USA). A calibration curve was plotted with the gallic acid standard, considering concentrations from 0.06 to 1 mg/mL (R^2^ = 0.998). The results were reported as mg of gallic acid equivalents per gram of sample (mg GAE/g DS).

The determination of flavonoid content was performed according to Del-Toro-Sánchez et al. [21]. A total of 80 μL of each sample and 80 μL of an ethanolic solution of AlCl_3_ (20 g/L) were added to wells of the microplate, shaken for 30 s, and left in the dark at room temperature for 1 h. Subsequently, it was shaken again for 30 s, and the absorbance was measured at 415 nm. A calibration curve was plotted with the quercetin standard at concentrations from 0 to 0.3 mg/mL (R^2^ = 0.998). The results were reported as mg of quercetin equivalents per gram of sample (mg EQ/g DS).

### 2.9. Antioxidant Capacity Essays

For the measurement of antioxidant capacity by scavenging 2,2-diphenyl-1-picrylhydrazyl radical (DPPH^•^), 1.5 mg of DPPH^•^ radical was dissolved in 50 mL of methanol. The absorbance of the solution was adjusted to 0.7 ± 0.01 at a wavelength of 515 nm. A total of 200 µL of the solution and 20 µL of extract and sample solutions were added to the microplate wells, left to rest in the dark for 30 min, and the absorbance was measured at a wavelength of 515 nm [22]. A calibration curve was plotted with the Trolox standard, considering concentrations between 0 and 0.08 mg/mL (R^2^ = 0.993). The results were expressed as µM Trolox equivalents per gram of sample (µM TE/g DS).

For the determination of antioxidant capacity by 2,2′-azino-bis (3-ethylbenzothiazoline-6-sulfonic acid) (ABTS^•+^) assay, 19.3 mg of ABTS were dissolved in 5 mL of distilled water. Separately, 0.0378 g of potassium persulfate and 1 mL of water were mixed. Subsequently, 88 µL of the potassium persulfate solution was added to the ABTS solution and left to rest in the dark for 12 h under refrigeration. A total of 1 mL of this last solution was added to 88 mL of ethanol, and the absorbance was adjusted to 0.7 ± 0.01 at a wavelength of 734 nm. For measurements, 270 µL of the adjusted solution and 20 µL of each sample were added to the microplate wells. The absorbance was measured at 734 nm in a microplate reader after 30 min of rest [23]. A calibration curve was plotted with the Trolox standard at concentrations from 0 to 0.1 mg/mL (R^2^ = 0.992). The results were expressed in µTrolox equivalents per gram of sample (µM TE/g DS).

To measure the ferric-reducing antioxidant power, three stock solutions were prepared: a sodium acetate buffer solution (300 mM/L at a pH of 3.6), a 20 mM FeCl_3_ × 6H_2_O, solution, and a 10 mM TPTZ in 40 mM HCl solution. The working solution was obtained by adding the three stock solutions in a 10:1:1 ratio (buffer/FeCl_3_ × 6H_2_O/TPTZ-HCl). For measurements, 20 µL of samples (extract of bitter orange peel, solutions of empty gelatin nanoparticles, and nanoparticles containing the extract) and 280 µL of the working solution were added to the microplate wells and the absorbance was measured at 638 nm after 30 min of rest. A calibration curve was plotted with the Trolox standard, considering concentrations between 0 and 1 mg/mL (R^2^ = 0.995). The results were expressed as µM Trolox equivalents per gram of sample [22].

### 2.10. Antimicrobial Activity

*Escherichia coli* O157:H7 (ATCC 43890) and *Listeria monocytogenes* (ATCC 7664) strains were used in this study. An aliquot of each strain was added to 3 mL of tryptic soy broth and left to incubate at 37 °C for 18 h. To obtain pure cultures, a loop of *E. coli* O157:H7 was streaked on Sorbitol MacConkey Agar and *L. monocytogenes* on Oxford Agar. The plates were left incubated at 37 °C for 24 h. For each bacterium, a colony from the plates was inoculated in 9 mL of trypticase soy broth [24]. The population of the suspension was adjusted to 1.5 × 10^8^ cells/mL using the McFarland standard. Serial decimal dilutions were performed to obtain inoculums with a population of 10^3^ CFU/mL. For the agar well diffusion method, 100 μL of each inoculum were streaked on plates with Mueller–Hinton Agar. Subsequently, three 6 mm wells were made per plate, into which 50 μL of each sample was added [25]. The plates were incubated for 18 h at 37 °C. The wells with 50 μL of ethanol were considered as negative control. After incubation, the inhibition zones were measured with a vernier caliper.

### 2.11. Statistical Analysis

The results of the antioxidant and antibacterial activity determinations were performed in triplicate and were presented as mean ± standard deviation. A one-way analysis of variance (ANOVA) and a Tukey test were carried out to determine whether there was a significant difference in the antioxidant and antibacterial activity of the extract of bitter orange peel, the empty gelatin nanoparticles, and the nanoparticles containing the extract (*p* < 0.05) [26]. The statistical analyses were performed using Minitab 17 (State College, PA, USA).

## 3. Results and Discussion

### 3.1. Preparation of Bitter Orange Peel Extract

After the ultrasound-assisted extraction process and the concentration of the extract by evaporation under reduced pressure, an average yield of 1.076% of ethanolic extract of bitter orange peel was estimated considering the two batches. A concentration of 70 mg/mL was chosen for electrospray nanoencapsulation and subsequent analyses.

### 3.2. Obtaining of Nanoparticles

**Figure 2:**
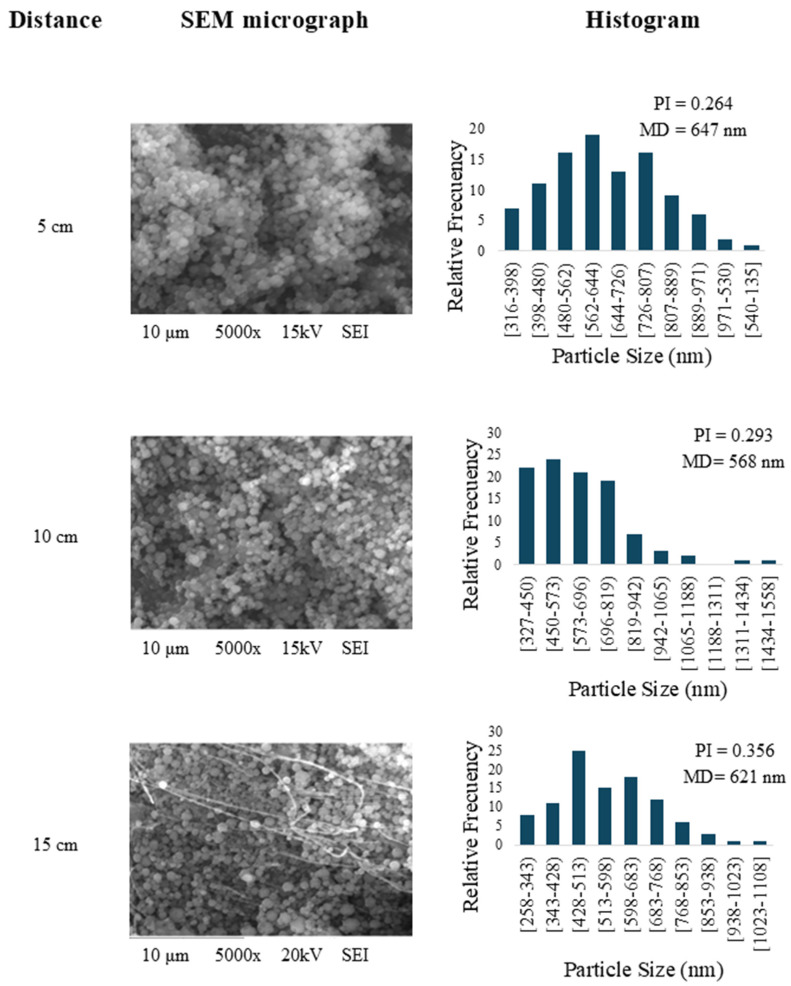
Preliminary tests for the preparation of gelatin nanoparticles using electrospray: SEM micrographs (5000× magnification) and histograms at collector distances of 5, 10, and 15 cm.

The determination of the best conditions of nanoencapsulation was based on the values of mean diameter (MD) and polydispersity index (PI) for the empty particles produced by uniaxial electrospray. This involved preparing a feed solution consisting of 10% *w*/*v* gelatin in 20% acetic acid, positioned at distances of 5, 10, and 15 cm from the collector. SEM micrographs indicate that the distance between the collecting plate and the needle significantly impacted the uniformity of the particles (Figure 2). Consequently, this led to the generation of more scattered particles with varying sizes, contributing to an increase in the PI. Considering the above, the most favorable condition for producing gelatin nanoparticles was achieved when the collector was positioned at 5 cm. On the contrary, Torkamani et al. [16] investigated different combinations of voltage (15, 20, and 25 kV) and flow rate (0.25, 0.5, and 0.75 mL/h) while maintaining a polymer concentration of 5% *w*/*w*. The authors found that the optimal conditions for producing spherical gelatin beads with an average diameter of 297 ± 70 nm were 20 kV, a flow rate of 0.5 mL/h, and 10 cm of collector distance. Therefore, the formation of spherical gelatin beads in the present study and the reported studies could be differentiated based on the experimental conditions. Torkamani et al. [16] utilized a higher voltage, a higher flow rate, a larger collector distance, and a lower polymer concentration than in our study.

### 3.3. Preparation of Gelatin Nanoparticles Containing an Extract of Bitter Orange Peel

**Figure 3:**
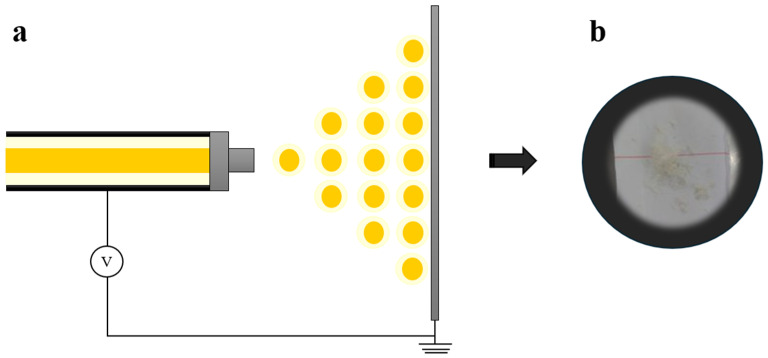
Coaxial electrospray process (**a**) and powder of gelatin nanoparticles containing bitter orange peel extract (**b**).

The ethanolic extract of bitter orange peel was encapsulated with bovine type B gelatin by coaxial electrospray, considering the previously established conditions (Figure 3). These materials were chosen because proteins, such as gelatin, are biocompatible and readily available as raw materials. It should be noted that there is currently no available data in the literature regarding the nanoencapsulation of an ethanolic extract of bitter orange peel using protein as an encapsulating agent. However, some authors synthesized buriti oil nanoparticles using water-in-oil emulsification with porcine gelatin alone and in combination with alginate. The particles formed with only gelatin were spherical, had a smooth surface, showed a homogeneous size distribution, exhibited low agglomeration, and were less than 100 nm in size [27].

**Figure 4:**
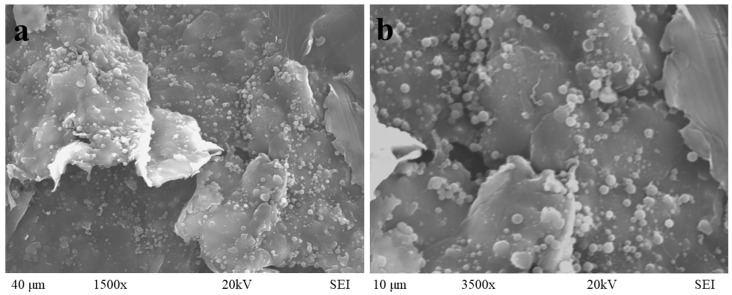
SEM micrograph of gelatin nanoparticles containing bitter orange peel extract. Magnification (**a**) 1500× and (**b**) 3500×.

The coaxial electrospray method employed a flow rate of 0.1 mL/h for the gelatin solution and 0.08 mL/h for the extract. The collection distance was set at 5 cm, operating under 15 kV, and the collection process lasted approximately 1 h. The resultant gelatin nanoparticles, incorporating bitter orange peel extract, exhibited a spherical shape and a uniform size, as depicted in Figure 4. This was confirmed by assessing the polydispersity index (PI) and mean diameter (MD), whose values were 0.435 and 0.675 µm, respectively. In a separate study, Figueroa et al. [26] utilized a 10% *w*/*v* gelatin solution to encapsulate betalains extracted from pitaya at concentrations of 1%, 3%, and 5% *w*/*v*. Interestingly, the mean diameter and polydispersity index of the nanoparticles did not vary significantly across these different betalains concentrations. Specifically, the mean diameter values were reported as 846, 832, and 839 nm, while the polydispersity index values were 0.12, 0.13, and 0.13, respectively. Discrepancies in PI values between studies can be attributed to differences in environmental conditions. In the present study, nanoparticle production occurred under humidity conditions ranging from 48% to 55% throughout the day, as efforts were made to identify optimal conditions for nanoparticle preparation. Furthermore, it was discovered that higher humidity levels corresponded to larger nanoparticle sizes [28].

**Figure 5:**
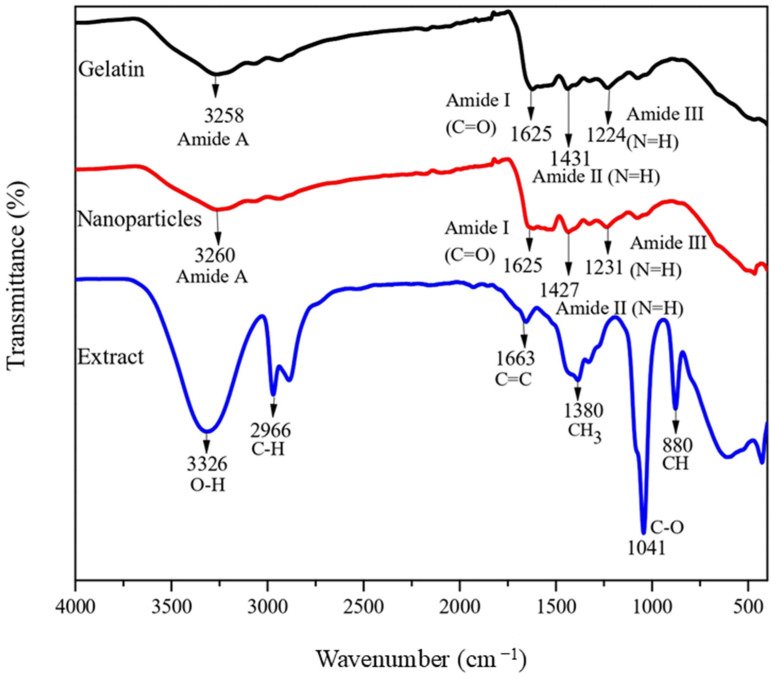
FT-IR spectra of gelatin, gelatin nanoparticles containing bitter orange peel extract, and ethanolic extract of bitter orange.

Figure 5 shows the infrared spectrum of the gelatin, gelatin nanoparticles containing bitter orange peel extract, and ethanolic extract of bitter orange peel. A total of 14 different bonds were identified, four of them characteristic of gelatin and typical of proteins: 3258 cm^−1^ (Amide A); 1625 cm^−1^ (Amide I), characteristic of the carbonyl bond (C=O); 1431 cm^−1^ (Amide II) associated with N-H bending; and 1224 cm^−1^ (Amide III), correlated with the vibrations of C-N and N-H bonds [29]. Four bonds were also observed for the gelatin nanoparticles containing orange peel extract: 3260 cm^−1^ (Amide A); 1625 cm^−1^ (Amide I), attributed to the carbonyl bond (C=O); 1427 cm^−1^ (Amide II), resulting from N-H bending; and 1231 cm^−1^ (Amide III), corresponding to the vibrations of C-N and N-H bonds. Additionally, other bonds were detected at specific frequencies: 3326 cm^−1^, representing O-H bond stretching and associated with phenols and carboxylic acids found in pectin and lignin; 2966 cm^−1^, indicating C-H bond stretching originated from methyl and methoxy groups; 1663 cm^−1^, indicating C=C bond stretching possibly due to the presence of benzenes or aromatic rings in carotenoids; 1380 cm^−1^, reflecting CH_3_ bending attributed to compounds such as hesperidin and carotenoids; 1041 cm^−1^, corresponding to the C-O group of alcohols and carboxylic acids found in bioactive compounds (ascorbic acid and naringin). Finally, a bond at 880 cm^−1^, corresponding to the C-H bond of aromatic compounds such as beta-carotene and phenols [30]. Encapsulating the bitter orange peel extract in the gelatin core did not significantly affect any of the characteristic amide bonds. This indicates that the inclusion of the extract did not alter the chemical structure of the gelatin and was a physical process [31].

### 3.4. Antioxidant Activity

As per Table 1, an assessment of total phenols, flavonoids, and antioxidant capacity was conducted for the ethanolic extract of bitter orange peel, gelatin nanoparticles, and gelatin nanoparticles containing ethanolic extract of bitter orange peel. The nanoparticles containing the extract showed the highest total phenol content (30.7 ± 3.02 mg EAG/g DS) compared to the extract (7.3 ± 0.59 mg EAG/g DS). This may occur because the technique used is sensitive to various compounds and bonds that can interact in the reactive system through electron transfer [32]. Conversely, the value obtained for the extract is closely similar to those found in the literature. Divya et al. [33] reported variations in the extraction of total phenols from lyophilized *Citrus aurantium* peel using different solvents: acetone (10.0 ± 0.70 ETA mg/g), hexane (5.0 ± 0.50 mg ETA/g), methanol (22.5 ± 0.80 mg ETA/g), ethyl acetate (5.0 ± 0.21 mg ETA/g), and water (45.0 ± 0.80 mg ETA/g). Therefore, the biological activity of the extract could depend on the extraction method and the solvent used.

The extract showed the highest flavonoid content (4.179 ± 0.012 mg EQ/g DS), presumably because when the flavonoids interact with gelatin, there may be alterations in the arrangement of hydroxyl groups. The substitution of hydroxyl groups by glycosylation could decrease the antioxidant activity [34]. On the other hand, it was noted that the addition of the ethanolic extract of bitter orange peel to the gelatin nanoparticles increased its activity of neutralizing radicals by DPPH^•^ and ABTS^•+^ essays (3.589 ± 0.534 and 65.671 ± 5.359 μM ET/g DS, respectively). There are few reports about the impact of the gelatin coating on antioxidant activity. However, bioactive compounds are protected by this gelatin protein layer. Moreover, the presence of a high concentration of antioxidant compounds acts through mechanisms involving hydrogen atom transfer to neutralize free radicals. For FRAP, the highest value was observed for the extract (180.607 ± 2.382 μM ET/g DS). This occurred because the antioxidant compounds responsible for neutralizing free radicals through electron transfer were less available in the gelatin nanoparticles, making it difficult for them to traverse the protein barrier and reduce the iron complex. Based on the findings, it can be deduced that, overall, gelatin nanoparticles serve as effective encapsulants, offering protection to the bioactive compounds found in the bitter orange peel.

### 3.5. Antimicrobial Activity

According to Table 2, greater inhibition of the Gram-negative bacteria (*E. coli*) was obtained with the gelatin nanoparticles containing the extract of bitter orange peel (1.967 ± 0.058 cm), possibly due to the phenolic compounds it presented. Electrostatic attractions, hydrophobic interactions, Van der Waals forces, and receptor–ligand interactions facilitate the adhesion between nanomaterials and microbial cells, resulting in the breakdown of the microorganism’s cell wall. Similarly, the destruction of the cell membrane is encouraged by the production of free radicals and reactive oxygen species (ROS), which compromise the antioxidant defense system and cause mechanical damage to the cell membrane. Following this, nanomaterials interact with key cellular organelles such as DNA, enzymes, ribosomes, and lysosomes. This interaction leads to various issues, including protein deactivation, oxidative stress, altered membrane permeability, heterogeneous changes, electrolyte imbalance, and modifications in gene expression [35].

The extract exhibited superior inhibition against Gram-positive bacteria (*L. monocytogenes*), with a diameter of inhibition measured at 1.833 ± 0.289 cm, owing to its elevated flavonoid content. Studies indicate that aglycone-type citrus flavonoids possess higher activity compared to glycosylated counterparts [36]. Additionally, the gelatin’s effect on the extract involves glycosylation, potentially leading to a reduction in both its antibacterial and antioxidant efficacy by replacing hydroxyl groups. In another research, gelatin nanoparticles containing buriti oil exhibited superior inhibition against Gram-negative bacteria *Pseudomonas aeruginosa* and *Klebsiella pneumoniae* compared to the Gram-positive microorganism *Staphylococcus aureus* [27].

## 4. Conclusions

Gelatin nanoparticles were prepared using coaxial electrospraying under specific conditions: a 5 cm distance between the needle and the collector, a pump flow rate of 0.1 mL/h for the polymer solution, and 0.08 mL/h for the ethanolic extract of bitter orange peel, with a voltage of 15 kV. The characterization of the nanoparticles containing an extract of bitter orange peel by SEM confirmed that they were monodisperse spheres of uniform size, while FT-IR analysis indicated that the incorporation of the extract into the gelatin nanoparticles consisted of a physical process. Additionally, it was found that encapsulation enhanced the antioxidant capacity by the DPPH^•^ and ABTS^•+^ assays. Both the extract and the nanoparticles containing extract showed effectiveness in inhibiting the Gram-negative microorganism *E. coli* O157:H7. Therefore, encapsulating the extract with gelatin provides significant benefits due to the protection of bioactive compounds for food applications.

## Figures and Tables

**Table 1:** Total phenolic and flavonoid content and antioxidant capacity of the ethanolic extract of bitter orange peel, the empty gelatin nanoparticles, and the nanoparticles containing the extract.

Determination	Ethanolic Extract of Bitter Orange Peel	Empty Gelatin Nanoparticles	Nanoparticles Containing the Extract of Bitter Orange Peel
Total phenols [mg EAG/g DS]	7.337 ± 0.586 ^b^	-	30.656 ± 3.015 ^a^
Flavonoids [mg EQ/g DS]	4.179 ± 0.012 ^a^	0.295 ± 0.042 ^b^	0.321 ± 0.085 ^b^
DPPH^•^ [μM ET/g DS]	1.246 ± 0.003 ^b^	1.885 ± 0.183 ^b^	3.589 ± 0.534 ^a^
ABTS^•+^ [μM ET/g DS]	17.171 ± 0.534 ^b^	13.238 ± 1.820 ^b^	65.671 ± 5.359 ^a^
FRAP [μM ET/ g DS]	180.607 ± 2.382 ^a^	62.916 ± 1.226 ^c^	112.431 ± 2.941 ^b^

Note: Distinct lowercase letters in the same row indicate a statistical difference (*p* < 0.05).

**Table 2:** Antimicrobial activity (zone of inhibition in centimeters) of the ethanolic extract of bitter orange peel, the empty gelatin nanoparticles, and the nanoparticles containing the extract.

Microorganism	Ethanolic Extract of Bitter Orange Peel	Empty Gelatin Nanoparticles	Nanoparticles Containing the Extract of Bitter Orange Peel
*E. coli* O157:H7	1.533 ± 0.321 ^a^	0.667 ± 0.058 ^b^	1.967 ± 0.058 ^a^
*L. monocytogenes*	1.833 ± 0.289 ^a^	0.020 ± 0.00 ^b^	0.700 ± 0.300 ^b^

Note: Distinct lowercase letters in the same row indicate a statistical difference (*p* < 0.05).

## Data Availability

The original contributions presented in the study are included in the article; further inquiries can be directed to the corresponding authors.
